# Glucose and Lipid Dysmetabolism in a Rat Model of Prediabetes Induced by a High-Sucrose Diet

**DOI:** 10.3390/nu9060638

**Published:** 2017-06-21

**Authors:** Ana Burgeiro, Manuela G. Cerqueira, Bárbara M. Varela-Rodríguez, Sara Nunes, Paula Neto, Frederico C. Pereira, Flávio Reis, Eugénia Carvalho

**Affiliations:** 1 Center of Neuroscience and Cell Biology (CNC) and CNC.IBILI Research Consortium, University of Coimbra, 3004-504 Coimbra, Portugal; burgeiroana@gmail.com (A.B.); manuela.g.cerqueira@gmail.com (M.G.C.); biobvr00@udc.es (B.M.V.-R.); sara_nunes20@hotmail.com (S.N.); fredcp@ci.uc.pt (F.C.P.); 2 Laboratory of Pharmacology and Experimental Therapeutics, Institute for Biomedical Imaging and Life Sciences (IBILI), Faculty of Medicine, University of Coimbra, 3000-548 Coimbra, Portugal; 3 Service of Anatomical Pathology, Coimbra University Hospital Centre (CHUC), 3000-075 Coimbra, Portugal; anahepafr@gmail.com; 4 The Portuguese Diabetes Association (APDP), 1250-203 Lisbon, Portugal; 5 Department of Geriatrics, University of Arkansas for Medical Sciences, Little Rock, AR 72202, USA; 6 Arkansas Children’s Hospital Research Institute, Little Rock, AR 72202, USA

**Keywords:** high-sucrose diet, prediabetes, glucose, lipid, metabolism, hypertriglyceridemia

## Abstract

Glucotoxicity and lipotoxicity are key features of type 2 diabetes mellitus, but their molecular nature during the early stages of the disease remains to be elucidated. We aimed to characterize glucose and lipid metabolism in insulin-target organs (liver, skeletal muscle, and white adipose tissue) in a rat model treated with a high-sucrose (HSu) diet. Two groups of 16-week-old male Wistar rats underwent a 9-week protocol: HSu diet (*n* = 10)—received 35% of sucrose in drinking water; Control (*n* = 12)—received vehicle (water). Body weight, food, and beverage consumption were monitored and glucose, insulin, and lipid profiles were measured. Serum and liver triglyceride concentrations, as well as the expression of genes and proteins involved in lipid biosynthesis were assessed. The insulin-stimulated glucose uptake and isoproterenol-stimulated lipolysis were also measured in freshly isolated adipocytes. Even in the absence of obesity, this rat model already presented the main features of prediabetes, with fasting normoglycemia but reduced glucose tolerance, postprandial hyperglycemia, compensatory hyperinsulinemia, as well as decreased insulin sensitivity (resistance) and hypertriglyceridemia. In addition, impaired hepatic function, including altered gluconeogenic and lipogenic pathways, as well as increased expression of acetyl-coenzyme A carboxylase 1 and fatty acid synthase in the liver, were observed, suggesting that liver glucose and lipid dysmetabolism may play a major role at this stage of the disease.

## 1. Introduction

Type 2 diabetes mellitus (T2DM) has become an epidemic of noncommunicable diseases, with 415 million people worldwide currently living with diabetes [1]. According to the International Diabetes Federation, about 5 million people died from DM in 2015 and the estimates indicate that there will be about 642 million people living with DM by 2040. In addition, there are about 318 million adults with impaired glucose tolerance (IGT), which puts them at high risk for the disease.

Prediabetes (or intermediate hyperglycemia) already displays metabolic alterations and is a high risk state for developing T2DM. According to the American Diabetes Association, prediabetes is distinguished by having impaired fasting glucose (IFG) (100–125 mg/dL glucose), IGT (140–199 mg/dL glucose 2 h after a 75-g oral glucose tolerance test), and glycated hemoglobin (HbA1c) levels between 5.7–6.4%. The prevalence of prediabetes is rapidly increasing with over 470 million people projected with prediabetes by 2030 [2]. This likely anticipates increased morbidity, mortality, and healthcare costs in the near future with DM management. Thus, preventing the progression of IGT and/or IFG to T2DM is the most rational and effective way to combat the DM epidemic and lessen healthcare costs. However, before we can succeed, we need to unravel glucose and lipid metabolism at this stage of the disease. 

Diets enriched in sugars including the intake of sugar-sweetened beverages have been consistently linked to the increased risk of hypertriglyceridemia, obesity, T2DM, and cardiovascular disease [3]. A central feature of T2DM is hyperglycemia, as a result of excessive hepatic glucose production, insulin resistance, and deficient secretion of pancreatic insulin. An often overlooked feature is the relationship between glucose and lipids, whose fluxes are indeed closely interlinked through the intersection of metabolic pathways at the acetyl-CoA formation [4]. Impairment of one pathway can indirectly have a major impact on another [4,5]. Molecular and metabolic abnormalities in insulin action, such as peripheral tissues (muscle, liver, and adipose tissues) insulin resistance, together with minor defects in insulin secretion, can be clearly identified before the development of obesity or hyperglycemia. These factors contribute to the increased fatty acid influx into the liver and muscle causing accumulation of toxic lipid metabolites. Particularly, chronically increased levels of plasma nonesterified fatty acids (NEFA) and triglyceride (TG)-rich lipoproteins impair lipid metabolism, a process referred to as lipotoxicity [6]. Furthermore, when the NEFA supply exceeds metabolic capacity, lipids accumulate in peripheral tissues, such as liver and muscle, inducing organ dysfunction [6]. Several recent lines of evidence show that the typical dyslipidemia in T2DM patients, characterized by elevated TGs, low high density lipoprotein cholesterol (HDL-c), and the predominance of small-dense low density lipoprotein (LDL) particles, may not only be the consequence of diabetes but may also cause disturbances of glucose metabolism. In fact, hypertriglyceridemia leads to elevated levels of free fatty acids (FFAs), which contribute to insulin resistance and β-cell dysfunction, putatively by impairing the molecular mechanisms linking insulin receptors with glucose transporters, as well as by directly damaging β-cells. Moreover, hypertriglyceridemia and elevated FFAs can contribute to inflammation, which boosts insulin resistance and β-cell dysfunction [4,5,6,7]. In addition, low HDL-c could also influence low-grade inflammation and affect glucose metabolism, thus contributing to diabetes [8]. Collectively, lipotoxicity and glucotoxicity are associated to the progression of DM and its micro- and macrovascular complications [5,9]. However, the nature of glucose and lipid deregulation in the prediabetic state remains to be elucidated.

Therefore, we aim to evaluate glucose and lipid metabolism in a prediabetic rat model, induced by a high-sucrose (HSu) diet [10,11], focusing on the main insulin-target organs: liver, skeletal muscle, and white adipose tissue.

## 2. Materials and Methods 

### 2.1. Animals and Diets

Male Wistar rats (16 week-old; Charles River Laboratories, Barcelona, Spain) were housed, two per cage, under controlled conditions (12 h light/dark cycle schedule and controlled temperature (22 ± 1 °C) and humidity). After an adaptation period of 1 week, rats were randomly divided into two groups (2 animals per cage) and submitted to a 9-week protocol: (1) control—receiving tap water as vehicle; (2) Hsu—receiving 35% sucrose (S0389; Sigma-Aldrich, St. Louis, MO, USA) in the drinking water. All animals were fed standard rat chow, containing 60% of carbohydrates, 16.1% of protein, 3.1% of lipids, 3.9% of fibers, and 5.1% of minerals (AO4 Panlab, Barcelona, Spain), ad libitum (except during fasting periods). Body weight, food, and beverage consumption were monitored every two days and presented as weekly variation per rat (which is the mean of the two rats per cage). All experiments were conducted in accordance with the European Union (EU) Legislation for the protection of animals used for scientific purposes “Animals (Scientific Procedures) Act”, Directive 2010/63/EU, and with the National and Local Authorities, under authorization of the Organ Responsible for Animal Welfare of Faculty of Medicine of Coimbra University (07/2016). 

### 2.2. Chemicals

Collagenase type II was from Roche (Lisbon, Portugal). D-[[^14^C(U)]-glucose (250 mCi/mmol/L) was from Scopus Research BV (Wageningen, The Netherlands). Human insulin, Actrapid, was kindly supplied by Novo Nordisk A/S (Paço de Arcos, Portugal). N-heptane was from Merck-&-Co., Inc. (Whitehouse Station, NJ, USA). Optiphase Hisafe was from PerkinElmer, Inc. (Waltham, MA, USA). RNeasy^®^ MiniKits were from QIAGEN Sciences (Germantown, MD, USA). High Capacity cDNA Reverse Transcriptase kits were from Applied Biosystems (Forest City, CA, USA). PCR primers were designed using Beacon Designer software and synthesized by IDT-Integrated DNA Technologies, Inc. (BVBA, Leuven, Belgium). SYBRGreen Supermix was from Quanta Biosciences (Gaithersburg, MA, USA). All other reagents were from Sigma (St. Louis, MO, USA). ECF reagent was from GE Healthcare (Little Chalfont, UK).

### 2.3. Metabolic Characterization

Metabolic characterization was performed as previously described [10] and is detailed in the supplemental material. This included a glucose tolerance test (GTT), an insulin tolerance test (ITT), fasting insulin levels, and insulin resistance (evaluated by the homeostatic model assessment of insulin resistance HOMA-IR index), as well as serum TG to HDL-c ratio (TG/HDL-c), TG-glucose (TyG) index, as well as fed and fasted alanine aminotransferase [6] and aspartate aminotransferase (AST) assessment. Caloric intake was calculated based on the daily food intake, and in HSu-treated animals, the energy related to sucrose consumption was also calculated.

### 2.4. Blood and Tissues Collection

After a 9-week treatment, blood and tissues (liver, skeletal muscle—from the posterior thigh of the rat leg—and epididymal adipose tissue) were snap frozen and stored at −80 °C. Details are available in the supplemental material. 

### 2.5. Liver Pathology and Triglyceride Content

Liver TG were extracted and quantified as previously reported [12]. Briefly, 50 mg of liver samples were homogenized in 0.5 mL of chlorophorm/methanol (2:1) and incubated for 3 h with agitation at 4 °C. 300 μL of milliQ water were then added to the homogenate and centrifuged (13,000 rpm for 20 min, room temperature). The organic phase was transferred to a clean Eppendorf tube, allowed to evaporate at 4 °C, and finally stored at −20 °C. Liver TG quantification was performed with a specific commercial kit from Spinreact. Colorimetric determination was performed in a spectrophotometer (SPECTRAmax PLUS384, Molecular Devices, Sunnyvale, CA, USA) at a wavelength of 505 nm or 490 nm after TG resuspension in 500 μL of chloroform. Intensity of the formed color was proportional to TG concentration in each sample. Oil red O staining of liver frozen sections was performed to evaluate lipid deposition, according to previously described methodology [13]. Liver pathology was assessed by hematoxylin and eosin (H&E) in paraffin-embedded sections fixed with paraformaldehyde at the moment of sample collection. Data analysis was performed in a blind fashion by a pathologist.

### 2.6. Cell Size and Weight, Glucose Uptake, and Lipolysis in Isolated Epididymal Adipocytes

Epididymal adipocyte size and weight, insulin-stimulated D-[[^14^C(U)]-glucose uptake, and isoproterenol-stimulated lipolysis in isolated adipocytes were performed as previously described [14,15]. Briefly, for the insulin-stimulated glucose uptake, the isolated adipocytes were diluted ten times and were stimulated or not with human insulin (1000 μU/mL), for 10 min, at 37 °C, in a shaking water-bath. Subsequently, 0.86 µM D-[[^14^C(U)]-glucose was added to the medium and the accumulation of glucose followed for 30 min. The cell suspension was then transferred to pre-chilled tubes, containing silicone oil, allowing cells to be separated from the buffer by centrifugation. Cell-associated radioactivity was determined by liquid scintillation counting, which allowed us to calculate the rate of transmembranar glucose transport, according to the formula: cellular clearance of medium glucose = (c.p.m. cells × volume)/(c.p.m. medium × cell number × time).

For isoproterenol-stimulated lipolysis, the isolated adipocytes were diluted ten times and were incubated in the presence or absence of insulin (1000 μU/mL), in a shaking water bath, at 37 °C, for 60 min. The medium was also supplemented or not with isoproterenol (1 µM). Following incubation, cells were separated from the medium by centrifugation and glycerol levels were measured in the medium using an assay kit (Zen Bio, Inc., Research Triangle Park, NC, USA). Further details are available in the supplemental material.

### 2.7. Liver, Skeletal Muscle, and Adipose Tissue Gene and Protein Expression 

Total RNA from liver, skeletal muscle, and epididymal adipose tissue was extracted, and cDNA synthesis and relative mRNA levels for glucose transporter-1(Glut1), -2 (Slc2a2), and -4 (Glut4), phosphoenolpyruvate carboxykinase (Pepck), glucose-6-phosphatase (G6pc), acetyl-CoA carboxylase 1 (Acc1), fatty acid synthase (Fasn), diglyceride acyltransferase (Dgat1), carbohydrate-responsive element-binding protein (Mlxipl/Chrebp), sterol regulatory element-binding transcription factor 1 (Srebf1), and hormone-sensitive lipase (Hsl) were measured (by real time-PCR), as previously described [16]. Protein extraction and Western blot analysis of GLUT1, GLUT2, GLUT4, PEPCK, G6PC, ACC1, FASN, DGAT1, ChREBP, SREBP, and HSL were performed as previously described [14,15]. The supplemental material details the protocols and lists the primer sequences (Table S1) and antibodies (Table S2) used.

### 2.8. Statistical Analysis

Results were expressed as mean ± standard error of the mean, using GraphPad Prism, version 6 (GraphPad Software, San Diego, CA, USA). Student’s *t*-test for normally distributed data or the Mann Whitney test for non-normally distributed data were performed when two groups were considered. One-way or two-way ANOVA, followed by Tukey post hoc test, for multiple comparisons, was used as appropriate. Repeated measures ANOVA, followed by the Tukey post-hoc test, was used to assess the differences between groups and between basal and insulin-stimulated glucose uptake. Differences were considered significant when * *p* ≤ 0.05, ** *p* ≤ 0.01, *** *p* ≤ 0.001, or **** *p* ≤ 0.0001.

## 3. Results

### 3.1. HSu Diet Increases Beverage Consumption and Caloric Intake, Maintaining Body Weight

**Figure 1:**
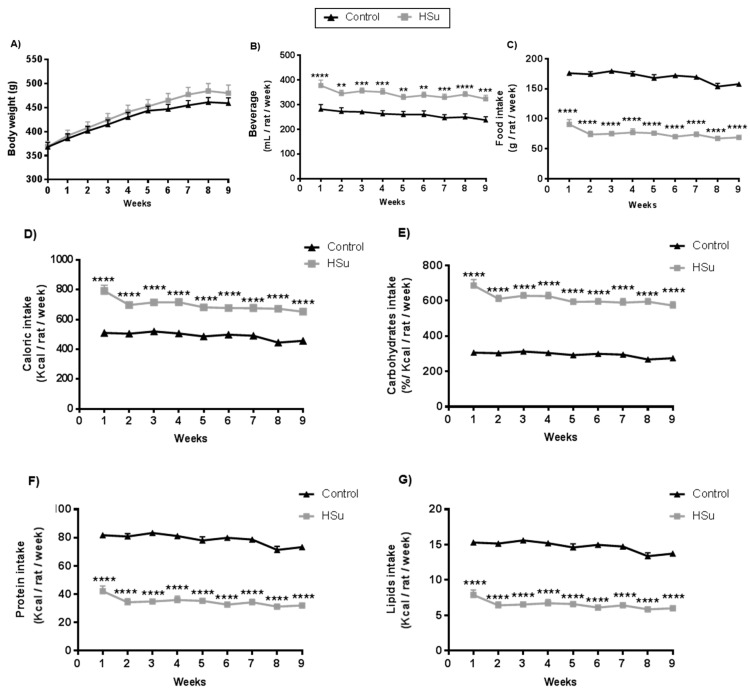
Evolution of body weight (**A**), beverage (**B**) and food (**C**) intake, as well as total caloric consumption (**D**) and caloric intake related with carbohydrates (**E**), protein (**F**), and lipids (**G**), throughout the 9 weeks of treatment. Control (*n* = 12) and HSu (*n* = 10). ** *p* < 0.01, *** *p* < 0.001, **** *p* < 0.0001.

During 9 weeks of treatment, the body weight evolution was identical in HSu-treated and control rats (Figure 1A). However, HSu-treated animals had a higher beverage (35% sucrose) consumption but a lower food ingestion (Figure 1B,C). This behavior translated to an increased total caloric intake, mainly from carbohydrates, with a lower calorie consumption from proteins and lipids, when compared with the control animals ((Figure 1D–G). 

### 3.2. HSu Diet Increases Serum Triglyceride Levels and Impairs Liver Function 

**Figure 2:**
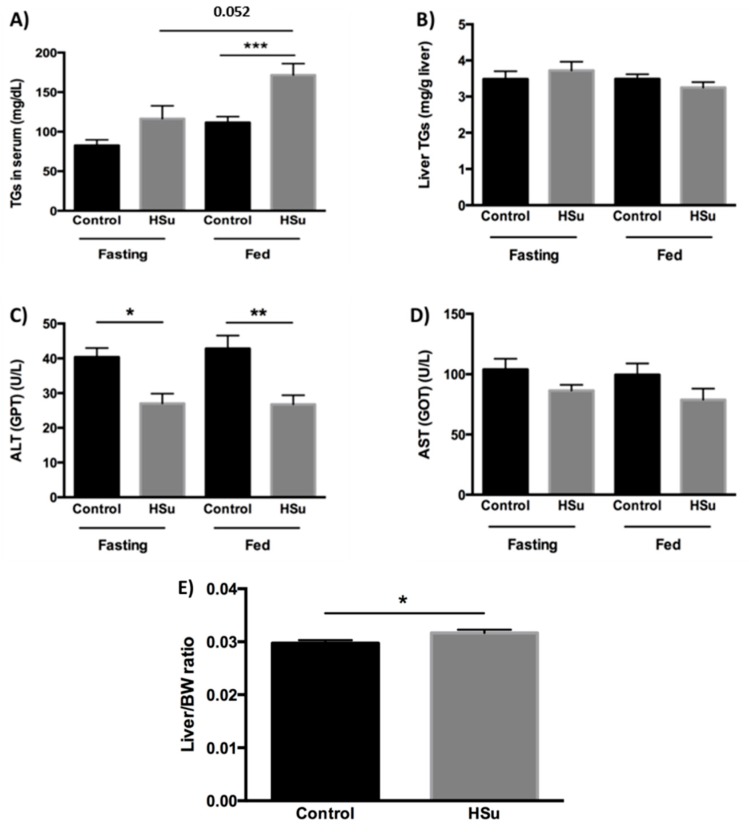
Serum (**A**) and liver (**B**) triglyceride (TG) content, serum alanine aminotransferase (ALT) (**C**) and aspartate aminotransferase (AST) (**D**) levels, as well as liver weight/body weight ratio (**E**) at the end of treatment. Control (*n* = 12) and HSu (*n* = 10). * *p* < 0.05, ** *p* < 0.01, *** *p* < 0.001.

The serum TG content in HSu-treated rats was significantly higher than in control animals in the fed state. Also, fed HSu-treated rats presented higher TG concentrations than in fasted HSu animals (Figure 2A). However, no significant differences were found in liver TG levels (Figure 2B). Regardless of the nutritional status (fasted or fed), serum ALT levels were reduced in the HSu-treated rats (Figure 2C); however, no significant differences were found in serum AST levels (Figure 2D). In addition, the liver weight/body weight ratio was increased in HSu-treated animals (Figure 2E). 

**Figure 3:**
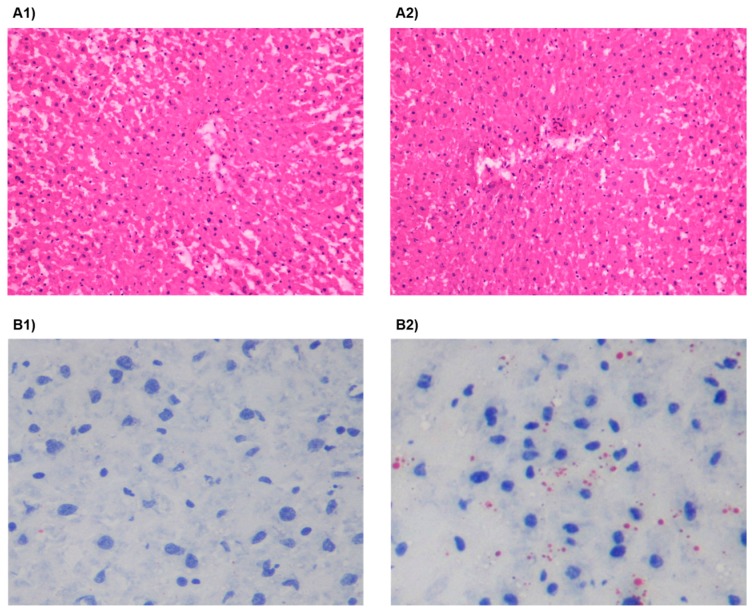
Liver (**A1**,**A2**) Hematoxylin and Eosin (H&E) staining (×200) and (**B1**,**B2**) Oil red O staining (×400) in control (1) and HSu-treated (2) rats. Six sections per group were measured.

Despite markers of impaired liver function, no changes in liver structure were found when analyzed by H&E histology (Figure 3(A1,A2)). Oil red O staining showed a slight deposition of lipids in the HSu-treated animals, when compared with the control ones (Figure 3(B1,B2)), respectively).

### 3.3. HSu Diet Impairs Glucose Tolerance and Causes Insulin Resistance

**Figure 4:**
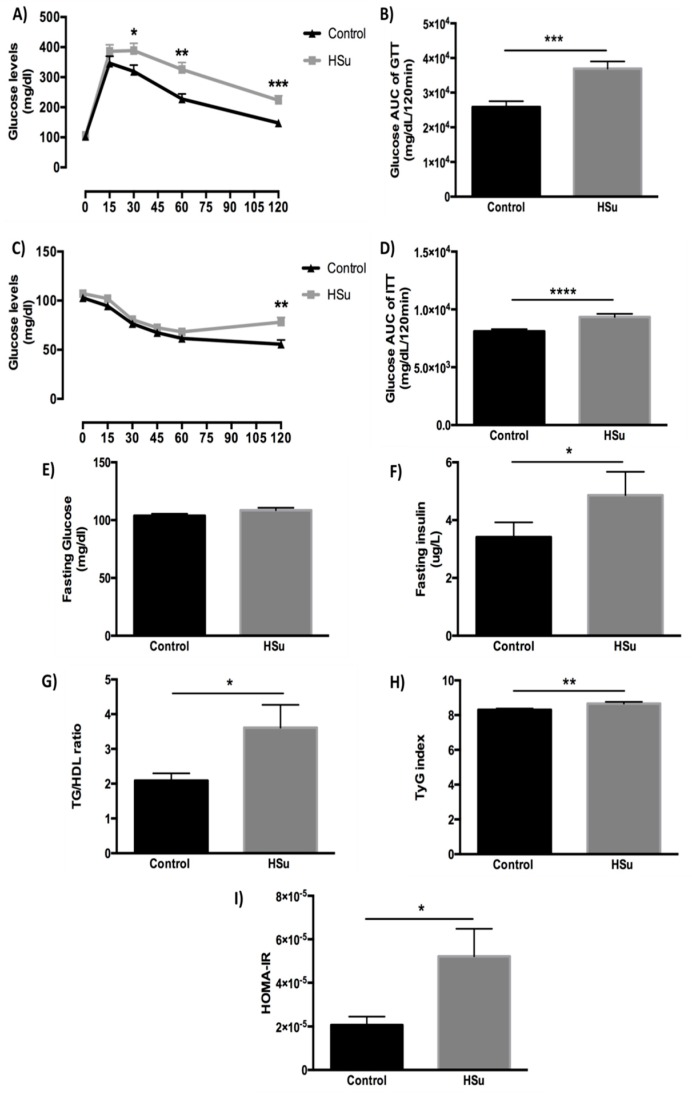
Glucose tolerance test (GTT) and area under the curve (**A**,**B**), insulin tolerance test (ITT) and area under the curve (**C**,**D**), fasting glucose (**E**) and insulin (**F**) levels, as well as insulin resistance markers: triglycerides to high density lipoprotein cholesterol (TG/HDL-c) ratio (**G**), triglyceride glucose (TyG) index (**H**) and homeostatic model assessment of insulin resistance (HOMA-IR) index (**I**) at the end of treatment. Control (*n* = 12) and HSu (*n* = 10). * *p* < 0.05, ** *p* < 0.01, *** *p* < 0.001, and **** *p* ≤ 0.0001.

Although both groups showed similar fasting glucose levels (Figure 4A,C,E), the HSu-treated rats had significantly slower glucose excursion during a GTT (2 g/kg body weight of glucose), compared to control animals (Figure 4A,B), revealing glucose intolerance. Blood glucose levels were significantly elevated in the HSu-treated rats, 120 min after an insulin injection (0.75 U/kg body weight of insulin) (Figure 4C,D). In addition, serum fasting insulin concentration was significantly elevated in HSu-treated animals (Figure 4F). Moreover, TG/HDL-c ratio, TyG and HOMA-IR indexes, known markers of insulin resistance, were significantly elevated in the HSu-treated rats, compared to the controls (Figure 4G–I).

### 3.4. HSu Diet Increases Fat Mass While the Insulin-Stimulated Glucose Uptake in Adipocytes Is Impaired

**Figure 5:**
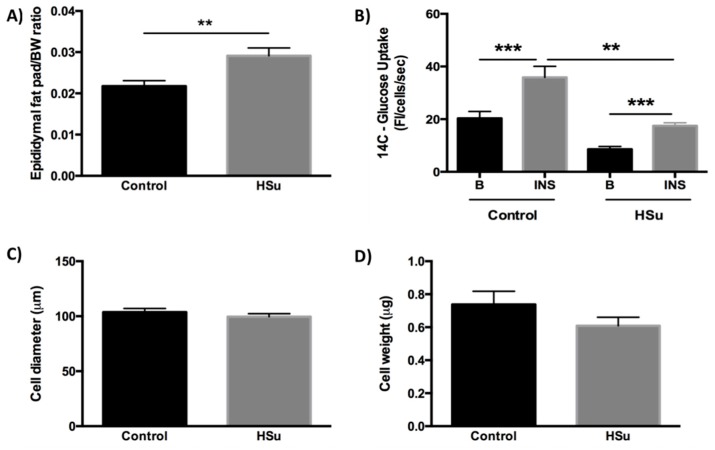
Epididymal fat pad weight/bw ratio (**A**), insulin-stimulated D-[[^14^C(U)]-glucose uptake in isolated adipocytes (**B**), and adipocyte diameter (**C**) and weight (**D**) at the end of treatment. Control (*n* = 12) and HSu (*n* = 10). ** *p* < 0.01, *** *p* < 0.001.

The epididymal fat pad weight/body weight ratio was significantly higher in the HSu-treated rats when compared to the controls (Figure 5A), while fat cell diameter and weight were unchanged between the groups (Figure 5C,D). Insulin-stimulated glucose uptake was measured in freshly isolated adipocytes. Basal glucose uptake was not significantly different between groups (*p* > 0.05). Adipocytes responded to the stimulatory effect of insulin in the uptake of glucose in both groups (Control − Basal = 20.31 ± 2.59 vs. Insulin = 35.88 ± 4.22, *p* < 0.001; HSu − Basal = 8.55 ± 1.09 vs. Insulin = 17.48 ± 1.16, *p* < 0.001); however, the insulin-stimulated glucose uptake was significantly reduced in adipocytes from HSu-treated rats compared to the controls (17.48 ± 1.16 vs. 35.88 ± 4.22, *p* < 0.01, respectively) (Figure 5B).

### 3.5. HSu Diet Decreased GLUT1 but Increased G6Pase Levels in Liver 

**Figure 6:**
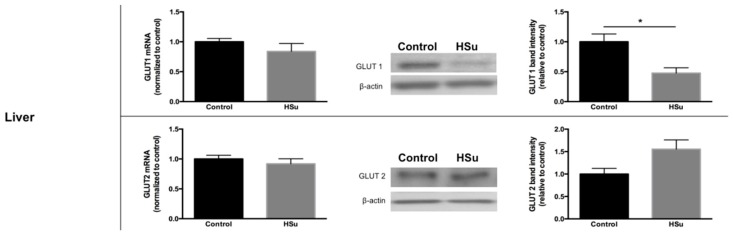

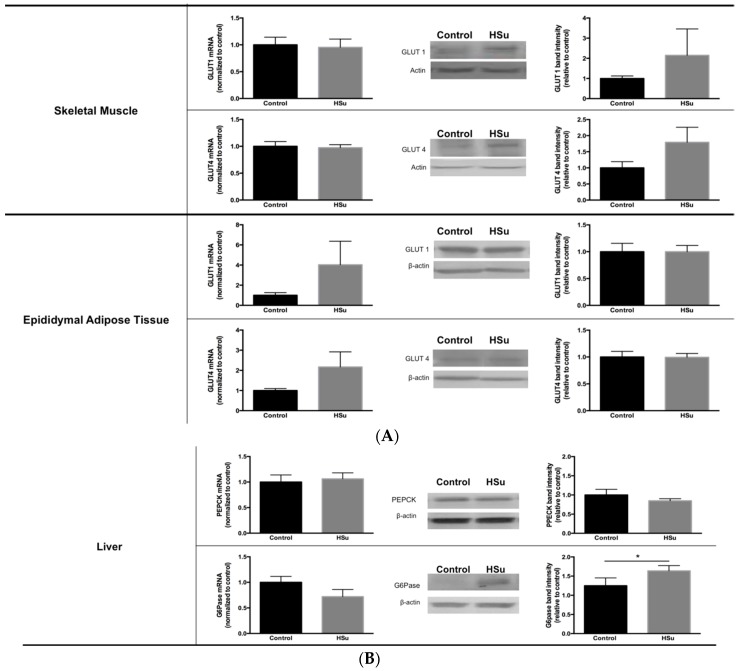
(**A**) Glucose transporters. (**B**) Gluconeogenesis. Gene and protein levels of glucose transporters (**A**) in liver, skeletal muscle (posterior thigh of the leg), and epididymal adipose tissue. Gene and protein levels of mediators of hepatic gluconeogenesis (**B**). Control (*n* = 12) and HSu (*n* = 10). * *p* ≤ 0.05.

The gene and protein levels of mediators of glucose uptake and gluconeogenesis were quantified in liver, skeletal muscle (posterior thigh of the leg), and epididymal adipose tissue. There were no changes in glucose transporter gene levels in all tissues, in the fed state (Figure 6A). Moreover, GLUT1 protein levels were reduced in HSu-treated rats versus controls, with no change in GLUT2 expression, in the liver. In addition, no differences were found in either GLUT1 or GLUT4 protein levels in skeletal muscle or epididymal adipose tissue. To assess hepatic gluconeogenesis, PEPCK and G6Pase gene and protein levels were evaluated. While there was no alteration in gene expression, there was a significant increase in only G6Pase protein levels in the HSu-treated group (Figure 6B).

### 3.6. HSu Diet Increases Hepatic Lipid Biosynthesis, without Alterations in Lipolysis

**Figure 7:**
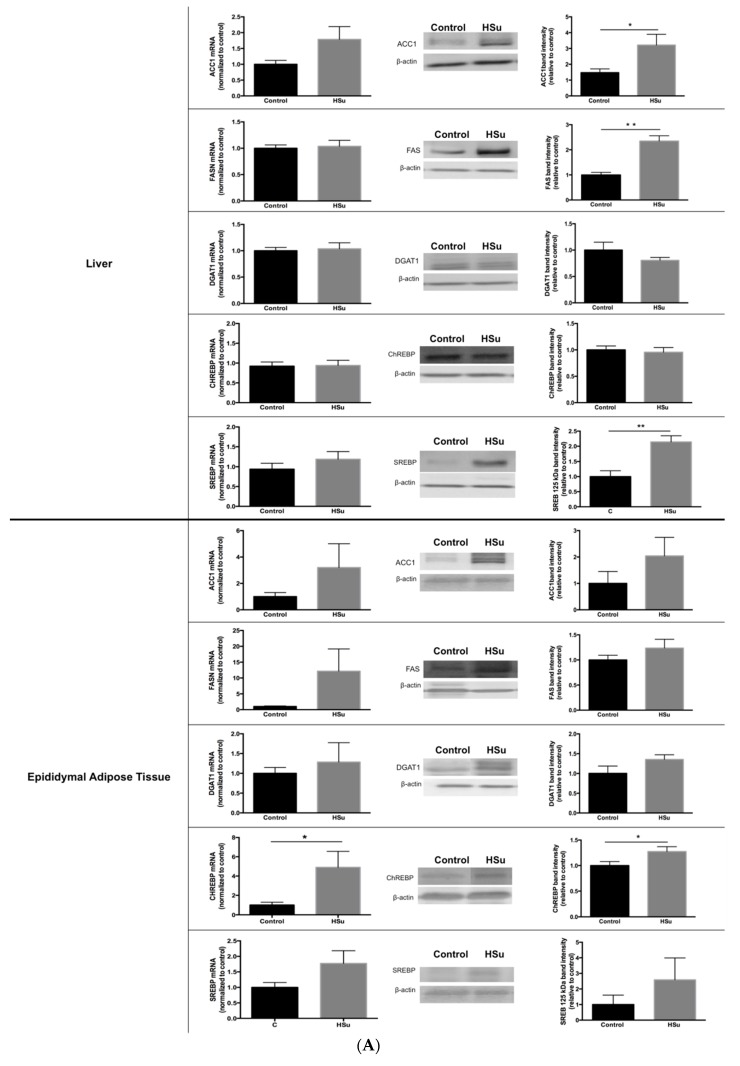

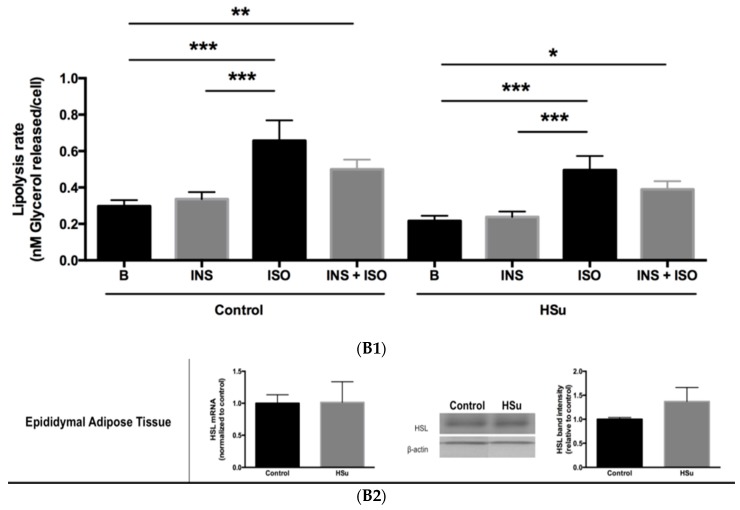
(**A**) Lipid biosynthesis. (**B1**,**B2**) Lipolysis. (**B1**) Isoproterenol-stimulated lipolysis. (**B2**) Hormone-sensitive lipase gene and protein. Gene and protein levels of mediators of lipid biosynthesis (**A**) in liver and epididymal adipose tissue. Isoproterenol-stimulated lipolysis (**B1**) and HSL gene and protein levels (**B2**) in epididymal adipose tissue. Control (*n* = 12) and HSu (*n* = 10). * *p* ≤ 0.05, ** *p* < 0.01, and *** *p* < 0.001.

Gene and protein levels of ACC1, FASN, and DGAT1, that play a major role in lipid biosynthesis in the liver and in fat, were analyzed, together with SREBP and ChREBP. Increased protein levels of ACC1, FASN, and SREBP were observed in the liver of HSu-treated animals, without changes in gene levels (Figure 7A). In addition, in adipose tissue, both gene and protein levels for ChREBP were increased in the HSu-treated rats, without further changes on the other measured mediators (Figure 7A).

Isoproterenol-stimulated lipolysis was performed to measure TG hydrolysis into glycerol and free fatty acids, and to evaluate the antilipolytic effect of insulin in isolated adipocytes (Figure 7(B1)). Both groups responded similarly to the stimulatory effect of isoproterenol (Control: 0.66 ± 0.11; HSu: 0.50 ± 0.08) versus basal (Control: 0.30 ± 0.03; HSu: 0.22 ± 0.03) and insulin levels (Control: 0.34 ± 0.04; HSu: 0.24 ± 0.03). Even though there is a tendency for an antilipolytic effect of insulin after isoproterenol stimulation (Control: 0.50 ± 0.05; HSu: 0.39 ± 0.05), it does not reach significance. Moreover, gene and protein expression levels for HSL were not different between the two groups (Figure 7(B2)).

## 4. Discussion

The novel findings from this study indicate that a high-sugar diet induces early glucose and lipid dysmetabolism, in the absence of weight gain, only after 9 weeks of treatment. HSu-treated animals developed impaired insulin-stimulated glucose uptake and reduced insulin sensitivity (resistance), together with decreased GLUT1 but increased G6Pase protein levels in the liver. These metabolic changes are paralleled by fed hyperglycemia and hypertriglyceridemia.

Throughout the 9-week treatment, the HSu-treated group had lower food consumption but higher beverage (35% sucrose) and caloric intake, while maintaining body weight, as previously described [17,18,19]. Similar body weight might have been maintained due to: first, differences in digestion and absorption may have modified the amount of ‘bioavailable’ energy, affecting the actual positive energy balance; second, the composition of weight gain (fat mass and lean mass) might be different, as the energy cost of protein deposition is higher than that of adipose tissue. In fact, we observed that HSu-treated animals have a greater adiposity translated by an increased epididymal fat pad weight/body weight ratio. Moreover, dietary protein content was suggested to be a critical determinant of weight gain during ad libitum feeding [20].

This prediabetic animal model presented low food intake, which is enriched in 16.1% protein, and increased fluid intake of sucrose-enriched water, in agreement with previous studies [18]. This means that HSu-treated animals ingested a smaller amount of protein and a greater amount of carbohydrates, which can be translated by higher fat mass and smaller lean mass, which allowed the maintenance of body weight. This suggestion might be further confirmed by performing an in vivo non-invasive ecographic magnetic resonance imaging (Echo-MRI) analysis to assess whole body fat, lean, free water, and total water masses.

Indeed, epididymal fat pad weight/body weight ratio was significantly higher in HSu-treated rats. Moreover, high-protein diets are known to reduce adiposity/lipogenesis in the context of high carbohydrate consumption in Western diets [21]. In fact, high-sucrose diets may increase adiposity by stimulating liver lipogenesis [22]. 

After 9 weeks of treatment, the HSu-treated group had higher serum TG levels, in agreement with previous reports [18,23,24,25], together with increased lipid deposition in the hepatic tissue, viewed by the Oil Red O staining. In addition, ALT levels were significantly reduced in HSu-treated rats, while a significant increase in the liver weight/body weight ratio was observed, suggesting altered liver function, without liver lesion at this stage of impaired metabolism. The strongest hypothesis to explain the reduced liver function and ALT low levels is malnutrition or an altered nutritional pattern. This prediabetes model had lower food consumption (constituted by 16.1% protein) and increased fluid intake (sucrose-enriched water). Thus, the low serum ALT levels, indicative of liver dysfunction, observed in the HSu-treated rats might be caused by a diet poor in essential nutrients/macromolecules (including proteins and lipids), i.e., malnutrition.

This prediabetes model presented impaired glucose homeostasis. Already at the early stages of the disease, there were initial metabolic deregulations, evidenced by alterations in glucose tolerance during the GTT and ITT, as well as the glucose and insulin levels, indicative of insulin resistance, in agreement with other studies [18,26,27]. At this stage, although fasting glucose levels are maintained within normal values (normoglycemia), due to compensatory hyperinsulinemia, there is already some degree of glucose intolerance and peripheral insulin resistance, in agreement with other reports [18,28,29,30].

Insulin resistance was demonstrated not only by the increased HOMA-IR index but also by augmented TG/HDL-c ratio and the TyG index, which have previously been used as surrogate measures of impaired insulin sensitivity in obese adolescents with normoglycemia, in prediabetes, as well as in T2DM [31]. 

In an attempt to explain the glucose, insulin, and lipid dysmetabolism in this model at the prediabetic stage, we assessed possible alterations in glucose and lipid metabolism in fat. Accordingly with previous studies [18], the HSu diet increased fat mass, which is closely linked to the development of severe peripheral IR and prediabetes [32,33]. Moreover, under the fed state, the HSu diet impaired insulin-induced glucose uptake in isolated adipocytes, confirming that visceral adiposity is strongly associated with impaired glucose uptake and IR [34]. Additionally, under IGT, adipocytes are resistant to insulin, and its effectiveness may be impaired, contributing to postprandial hyperglycemia in prediabetic states. Furthermore, the decrease in hepatic GLUT1 in the HSu-treated rats might be mediated by the hyperinsulinemia observed in this animal model [35], although supplementary confirmation of this hypothesis could be achieved by evaluating GLUT1 expression under fasting conditions. On the other hand, both GLUT2 (in the liver) and GLUT4 protein levels (in the adipose tissue and skeletal muscle), were unchanged. The translocation of both GLUT2 and GLUT4 to the plasma membrane is mediated by insulin; however, under IR conditions, this process is impaired [36,37], leading to decreased insulin-stimulated glucose uptake in hepatocytes (mostly by GLUT2), while increasing postprandial blood glucose levels. Even though we did not measure translocation of glucose transporters in this study, it may be possible that these processes are impaired, thus also contributing to the increased postprandial blood glucose levels. 

Another factor that may also be contributing to the postprandial hyperglycemia observed in this animal model is that high sucrose consumption increased gluconeogenesis, as previously reported [38]. However, under IGT and/or IR conditions, the derangement of hepatic glucose handling, indicated by changes in GLUT1 and G6Pase may at least in part lead to postprandial hyperglycemia in prediabetic states [39]. Furthermore, in this animal model of IR, insulin may not inhibit the de novo glucose production by the liver, leading therefore to elevated gluconeogenesis, resulting in elevated blood glucose levels in the fed state, as previously reported [35], as well as altered glucose tolerance during a GTT.

Furthermore, the HSu diet increased liver lipid biosynthesis. Our results show that ACC1 and FASN enzymes, as well as the transcription factor SREBP expression levels were increased in the liver of HSu-treated rats, in agreement with previous studies [40,41,42,43,44]. The perturbed liver lipid metabolism that was observed might explain the hypertriglyceridemia present in this prediabetic animal model. In addition, high sucrose consumption may also be contributing to hypertriglyceridemia due to the increased ChREBP gene and protein levels observed in fat [45]. However, the HSu diet did not induce changes in the isoproterenol-stimulated lipolysis in isolated adipocytes, and the HSL protein levels were not different. HSL lipolytic activity is regulated by reversible phosphorylation on five critical residues [46]. Therefore the absence of alterations in lipolysis suggest that HSL phosphorylation was not changed by the HSu diet. Finally, insulin did not show a significant antilipolytic effect at these insulin concentrations. However, it is important to note that we used supra-physiological insulin concentrations (1000 μU/mL), in order to evoke a sustained respond to study the impact of the treatment. Testing physiological insulin concentrations are warranted in both control and HSu animals in future research. In addition, there are other aspects for the forthcoming studies that could consolidate this model, namely lipid profiling of FPLC-separated lipoprotein fractions, quantification of FFA levels, as well as the estimation of VLDL secretion. Furthermore, the impact of the HSu diet on leptin serum levels and on leptin receptor expression would be of interest.

**Figure 8:**
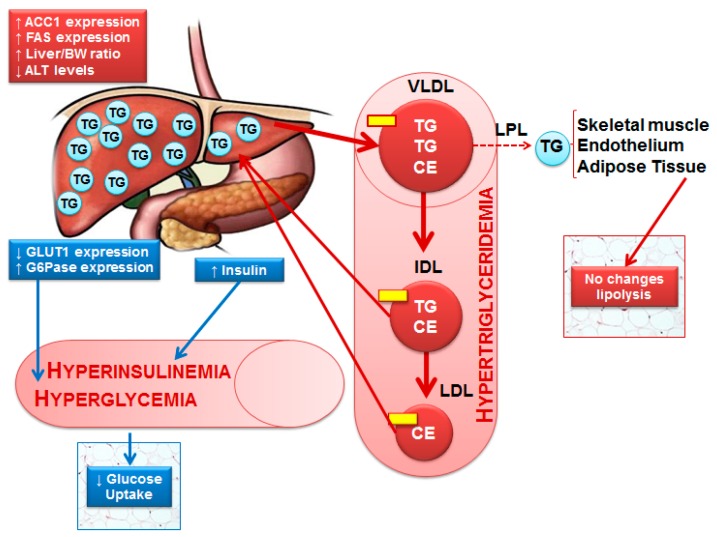
A molecular model describing insulin resistance and prediabetes development after a HSu diet. HSu diet increases serum triglyceride (TG) accumulation and triggers liver lipogenesis, inducing hypertriglyceridemia and interfering with liver function. However, no alterations were observed in lipolysis. The HSu diet alters hepatic proteins involved in basal glucose uptake and gluconeogenesis and impairs the insulin-stimulated glucose uptake in adipocytes, leading to impaired glucose homeostasis and increased IR markers. Legend: 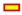 Apolipoprotein; CE—Cholesterol ester; IDL—Intermediate-density protein; LDL—Low-density lipoprotein; LDL—Lipoprotein lipase; TG—Triglycerides; VLDL—Very low-density lipoprotein.

Overall, our study shows that nine week of HSu-diet, which mimics at least in part Western diets [10,11,47], can lead to impaired hepatic glucose and lipid metabolism, typical features of T2DM, already present in this prediabetic model, even in the absence of obesity. This animal model of diet-induced prediabetes shows reduced glucose tolerance, postprandial hyperglycemia, hyperinsulinemia, reduced insulin sensitivity (resistance), and hypertriglyceridemia, together with impaired gluconeogenesis and lipogenesis (Figure 8).

This study should be viewed as an important wake up call for the lifestyle that the general population have been gradually adopting, by consuming large amounts of simple sugars in soft drinks [48]. Importantly, exaggerated consumption of these drinks has been associated with an increased risk for T2DM development by about 26% if the average intake is one/two cans a day, or even more [49]. This feeding behavior is one of the main causes of the uncontrolled increase of IR, T2DM, and associated complications, such as coronary heart disease [50,51]. 

## 5. Conclusions

This animal model of prediabetes induced by high-sucrose consumption presents liver glucose and lipid dysmetabolism. It is expected that new insights of impaired metabolism at this early stage of the disease might contribute to disclosing new therapeutic targets and strategies to counteract prediabetes and hinder the natural course of T2DM progression.

## Supplementary Materials

The following are available online at www.mdpi.com/2072-6643/9/6/638/s1, Supplemental methods; Table S1: Primer sequences for RT-PCR; Table S2: Antibodies used for Western blotting.
