# Supplementary material for: Glucose and Lipid Dysmetabolism in a Rat Model of Prediabetes Induced by a High-Sucrose Diet
Source: Nutrients. 2017 Jun 21;9(6):638. doi: 10.3390/nu9060638 (PMC5490617; doi:10.3390/nu9060638)
Supplement: Supplementary file 1 [file nutrients-09-00638-s001.zip › nutrients-191245-supplementary-.pdf]

nutrients

MDPI

1. SUPPLEMENTARY METHODS

Metabolic characterization: glucose, insulin and lipid assays

Metabolic characterization including the glucose tolerance (GTT) and the insulin tolerance tests (ITT), fasting insulin level measurement, insulin sensitivity assessment (HOMA index) were performed as we previously reported [5]. The triglyceride/high-density lipoprotein (TG/HDL) ratio and the triglyceride glucose (TyG) index were also assessed. Seric triglycerides, alanine and aspartate aminotransferase (ALT and AST, respectively) were assessed by automatic validated methods (Hitachi 717 analyser, Roche Diagnostics Inc., MA, USA) as previously reported [5].

The formulas were as follows: HOMA-IR=fasting serum glucose (mg/dL) × fasting serum insulin (μU/mL)/22.5; TG/HDL ratio=fasting triglycerides (mg/dL)/ fasting HDL (mg/dL); TyG index=(Ln[fasting triglycerides (mg/dL) × fasting glucose (mg/dL)/2]). The used values (insulin, glucose, triglycerides and HDL) were obtained after an overnight fast.

Blood and tissues collection

Blood samples were collected with rats under anesthesia (intraperitoneal) with a 2 mg/kg BW of a 2:1 (v:v) 50 mg/mL ketamine (Ketalar®, Parke-Davis, Lab. Pfeizer Lda, Seixal, Portugal) solution in 2.5% chlorpromazine (Largactil®, Rhône-Poulenc Rorer, Lab. Vitória, Amadora, Portugal). Blood samples were collected by venipuncuture, from the jugular vein, into Vacuette® (Greiner Bio-One GmbH, Germany) tubes without anticoagulant (to obtain serum) or with K3EDTA biochemical studies. Aliquots of serum and plasma were immediately stored at -80ºC until assayed. Animals were sacrificed by cervical dislocation and epididymal adipose tissue, liver and skeletal muscle samples were immediately removed, frozen in dry ice and stored at -80ºC for gene expression and Western-blot analysis.

Cell size and weight, glucose uptake and lipolysis in isolated epididymal adipocytes

Epididymal adipose tissue biopsies were cut into small pieces and digested with collagenase type II from Clostridium histolyticum in 6 mM glucose KHR buffer (4% bovine serum albumin (BSA), 140 mM sodium chloride (NaCl), 4.7 mM potassium chloride (KCl), 1.25 mM magnesium sulfate (MgSO4), 1.26 mM calcium chloride (CaCl2), 5.8 mM sodium phosphate (NaH2PO4), 200 nM adenosine deaminase and 25 mM HEPES, pH 7.4, adjusted with NaOH), for 30 min at 37º C, in a shaking water bath. Adipocyte size and weight were then determined as previously reported [8,10].

Insulin-stimulated D-[U-14C] glucose uptake in isolated adipocytes was assessed as previously reported [8]. Briefly, cell suspension was isolated from the undigested tissue by filtration through a 250-μm nylon mesh and washed four times in KHR medium without glucose. Then, isolated adipocytes were diluted ten times in KRH buffer without glucose and stimulated or not with human insulin (1000 μU/ml), during 10 minutes, in a shaking water-bath. After this pre-stimulation, 0.86 μM D-[14C(U)] glucose was added to the medium and the accumulation of glucose was followed for 30 minutes. After glucose incubation, the cell suspension was transferred to pre-chilled tubes, containing silicone oil, allowing the cells to be separated from the buffer by centrifugation. Cell-associated radioactivity was determined by liquid scintillation counting, which allowed us to calculate the rate of transmembranar glucose transport, according to the following formula: cellular clearance of medium glucose = (c.p.m. cells x volume)/(c.p.m. medium x cell number x time).

Lipolysis was also performed as previously reported [9]. Briefly, adipocyte suspension was incubated in the presence or absence of insulin (1000 μU/ml) in KRH buffer containing 6 mM glucose, in a gently shaking water bath at 37º C for 60 min. Also, the medium was supplemented or not with isoproterenol (1 μM). The medium was then separated from the adipocytes by centrifugation and glycerol levels were measured in extracellular medium using an adipocyte lipolysis assay kit (Zen Bio, Inc.); this was to estimate lipolysis in isolated epididymal adipocytes.

Liver, skeletal muscle and adipose tissue gene expression

Total RNA from liver, skeletal muscle and epididymal adipose tissue was extracted. The total RNA concentration was determined by OD260 measurement using the NanoDrop spectrophotometer (Thermo Scientific, USA). cDNA synthesis was performed using the High Capacity cDNA Reverse Transcriptase kit (Applied Biosystems; Forest City, CA, USA). Relative mRNA levels were measured by RT-PCR using specific primers for each target mRNA and Sybrgreen PCR mix (Quanta Biosciences, Inc., Gaithersburg, MA, USA) with a CFX ManagerTM version 2.0 Real-Time PCR detection system (Bio-Rad Laboratories, Hercules, CA, USA). Quantitative RT-PCR results were analyzed using delta CT calculations. Relative mRNA levels for the following genes – glucose transporter-1(Glut1), -2 (Slc2a2) and -4 (Glut4), phosphoenolpyruvate carboxykinase (Pepck), glucose-6-phosphatase (G6pc), acetyl-CoA carboxylase 1 (Acc1), fatty acid synthase (Fasn), diglyceride acyltransferase (Dgat1), carbohydrate-responsive element-binding protein (Mlxipl/Chrebp), sterol regulatory element-binding transcription factor 1 (Srebf1) and hormone-sensitive lipase (Hsl) – were determined and normalized using TATA-binding protein (Tbp) mRNA levels. All primers sequences are shown in Table 1 of supplemental material.

Liver, skeletal muscle and adipose tissue protein levels

25 mg of liver, 50 mg of skeletal muscle, and 200 mg of epididymal adipose tissue were homogenized in 550 µl of ice-cold RIPA buffer [20 mM Tris HCl pH 7.4, 25 mM NaCl, 1% NP-40 (Nonidet P-40), 5 mM EDTA, 10 mM Sodium diphosphate (Na4P2O7), 10 mM Sodium Fluoride (NaF), 2 mM Sodium Vanadate (Na3VO4), 10 µg ml-1 Aprotinin from bovine lung, 1 mM Benzamidine and 1 mM Phenylmethylsulfonyl fluoride (PMSF)] for western-blot analysis. The homogenized cells were placed on ice for about 30 min and, then, centrifuged at 17 000 rotations per minute (rpm) at 4°C for 10 min. The supernatant was subsequently collected and centrifuged at 17 000 rpm at 4°C for 10 min. The lower phase was then collected and protein concentration was determined using the bicinchoninic acid (BCA) method. Cell lysates were stored at -80ºC until further use. Cell lysates were denatured at 95 ºC, for 5 min, in sample buffer (Tris HCl 0.5 M 0.4% SDS (pH 6.8); 0.6 M DTT; 30% (v/v) glycerol, 10% SDS (w/v) and 0.01% bromophenol blue). 40 µg of protein were loaded on a 7.5% (v/v) sodium dodecyl sulfate polyacrylamide gel (SDS-PAGE) and then transferred to a polyvinylidene fluoride (PVDF) membrane. Membranes were blocked with TBS (50 mM Tris.HCl, pH 7.4, 150 mM NaCl) with 0.01 % of Tween 20 (TBS-T, pH 7.4) containing 5% dry milk for 1h at room temperature and then incubated overnight, at 4ºC, with primary antibodies (Table 2 of supplemental material). Membranes were, then, washed with TBS-T and incubated for 1h at room temperature with the corresponding alkaline phosphatase-conjugated secondary antibody (Table 2 of supplemental material). Membranes were subsequently washed with TBS-T, exposed to the ECF reagent and scanned by a VersaDocTM Imaging System (Bio-Rad Laboratories, Amadora, Portugal). Densitometric analyses were performed using Quantity OneTM Software. Actin antibody (Table 2 of supplemental material) was used as loading control.

2. SUPPLEMENTARY TABLES

Table S1. Primer sequences for RT-PCR.

Protein name	Gene	Primer Sequences	
**TATA box**	*TBP*	Forward	ACCCCACAACTCCTCTTCCATTC
		Reverse	CAAGTTTACAGCCAAGATTCA
**Glucose transporter 1**	*GLUT1*	Forward	TGCAGTTCGGCTATAACACC
		Reverse	CCCACAGAGAAGGAACCAATG
**Glucose transporter 2**	*SLC2A2*	Forward	AAGACAAGATCACCGGAACC
		Reverse	GACAGAGACCAGAGCATAGTC
**Glucose transporter 3**	*GLUT4*	Forward	CGTCATTGGCATCTCGGTTG
		Reverse	CTTTAGACTCTTTCGGGCAGG
**Phosphoenolpyruvate carboxylase**	*PEPCK*	Forward	ATCACCAACCCCGCAGGGAAA
		Reverse	TTGGATGCGCACAGGGTTCCT
**Glucose-6-phosphatase**	*G6PC*	Forward	GTGAATTACGAAGACTCCCAG
		Reverse	TGTTTTATCAGAGGCACGGAG
**Acetyl-CoA carboxylase 1**	*ACC1*	Forward	AAGGCTATGTGAAGGATGTGG
		Reverse	GAGGTTAGGGAAGTCATCTGC
**Fatty acid synthase**	*FASN*	Forward	GTGGAAGACACTGGCTCGAA
		Reverse	TGGTACACTTTCCCGCTCAC
**Diacylglycerol acyltrasferase 1**	*DGAT1*	Forward	GACAGGGGTTTCAGCAATTAC
		Reverse	GGGTCCTTCAGAAACAGAGAC
**Carbohydrate response element binding protein**	*MLXIPL*	Forward	CTTATGTTGGCAATGCTG
		Reverse	GGCGATAATTGGTGAAGA
**Sterol regulatory element-binding protein-1**	*SREBF1*	Forward	CGCTACCGTTCCTCTATCAATG
		Reverse	TCAGCGTTTCTACCACTTCAG
**Hormone-sensitive lipase**	*HSL*	Forward	GGGCAGAAGGATGAAACC
		Reverse	GACACAGAGGTAGAACTTGG

Table S2. Antibodies used for Western blotting.

Primary Antibodies	Dilution	Company	Expected molecular weight
**GLUT1**	1:1000 in 0.1% TBS-T with 5% BSA	Millipore	54 KDa
**GLUT2**	1:2000 in 0.1% TBS-T with 5% BSA	Millipore	57 KDa
**GLUT4**	1:1000 in 0.1% TBS-T with 5% BSA	Santa Cruz Biotechnology	54 KDa
**PEPCK**	1:2000 in 0.1% TBS-T with 5% Milk	Santa Cruz Biotechnology	62 KDa
**G6Pase**	1:2000 in 0.1% TBS-T with 5% Milk	Santa Cruz Biotechnology	36 KDa
**ACC1**	1:1000 in 0.1% TBS-T with 5% BSA	Cell Signaling	265 KDa
**FASN**	1:1000 in 0.1% TBS-T with 5% BSA	Cell Signaling	273 KDa
**DGAT1**	1:200 in 0.1% TBS-T with 5% Milk	Santa Cruz Biotechnology	55 KDa
**ChREP**	1:500 in 0.1% TBS-T with 5% Milk	Santa Cruz Biotechnology	62, 78, 91 and 93 KDa
**SREBP**	1:1000 in 0.1% TBS-T with 5% Milk	Santa Cruz Biotechnology	125 KDa
**HSL**	1:500 in 0.1% TBS-T with 5% BSA	Cell Signaling	81, 83 KDa
**β-actin**	1:5000 in 0.1% TBS-T with 1% BSA	Sigma-Aldrich	42 KDa
**Actin**	1:1000 in 0.1% TBS-T with 5% Milk	Santa Cruz Biotechnology	43 KDa
